# Improving diagnostic accuracy of ovarian torsion using contrast‐enhanced ultrasound: A prospective comparative clinical study of performance diagnosis

**DOI:** 10.1002/ijgo.70429

**Published:** 2025-08-05

**Authors:** Anne‐Laure Fijean, Gabriela Hossu, Aboubaker Cherifi, Marine Beaumont, Olivier Morel, Charline Bertholdt

**Affiliations:** ^1^ Obstetrics and Gynecology Department, CHRU Nancy University of Lorraine Nancy France; ^2^ Inserm, CIC, Innovation Technologique, CHRU Nancy University of Lorraine Nancy France; ^3^ INSERM U1254, Adaptive Diagnostic and Interventional Imaging University of Lorraine Nancy France

**Keywords:** acute abdominal pain, adnexal torsion, contrast‐enhanced ultrasound, functional imaging, gynecologic emergency, imaging diagnosis, ovarian cyst, ultrasound imaging

## Abstract

**Objective:**

This study evaluates the diagnostic performance of contrast‐enhanced ultrasound (CEUS) for improving diagnosis of adnexal torsion in women with acute abdominal pain.

**Methods:**

This was a pilot study of the diagnostic accuracy of CEUS for the detection of adnexal torsion. All women in our center with planned surgery for suspicion of adnexal torsion were included. The final diagnosis was confirmed during surgical intervention to classify “torsion” and “control” groups. Qualitative imaging analyses were performed blindly of the final diagnosis by an expert and nonexpert obstetrician and gynecologist (Obgyn).

**Results:**

Six ovaries were included in the torsion group and eight ovaries in the control group. A total of 476 images from 14 included cases were analyzed by one expert and 34 ObGyn. The sensitivity was 100% (confidence interval [CI] 95% [59–100]) and 84.3% (CI 95% [78.7–88.9]) and the specificity 86% (CI 95% [42–100]) and 89.8% (CI 95% [85.4–93.2]) for expert and nonexpert Obgyn, respectively.

**Conclusion:**

The diagnostic performance of CEUS to diagnose adnexal torsion is high.

## 1 INTRODUCTION

Adnexal torsion is due to total or partial rotation of the adnexa around its vascular axis, which is responsible for ischemia. The risk of oophorectomy and then impaired fertility, a consequence of ovarian necrosis, increases with the duration of ischemia and is often related to management delay.^1^, ^2^ It is therefore a surgical emergency with the aim of preserving the ovary and subsequent fertility.^3^, ^4^ However, diagnosis is challenging due to various clinical symptoms and the poor contribution of pelvic ultrasound, with sensitivity and specificity varying depending on ultrasound signs from 53% to 69% and 46% to 95%, respectively.^5^, ^6^ Other imaging techniques, such as magnetic resonance imaging, have been considered, but their accuracy and accessibility in the context of emergencies makes them difficult to use in clinical practice.^7^ Thus, surgical exploration is recommended in a short timeframe in cases of suspicion of adnexal torsion based on a range of clinical and ultrasound arguments.^8^ However, diagnosis was not confirmed surgically in 25–60% of cases in recent studies in women,^9^, ^10^, ^11^ and the rate of oophorectomy remains high and is probably related to management delay in confirmed cases.^12^, ^13^ Therefore, it is essential to improve the diagnostic accuracy of adnexal torsion to avoid unnecessary surgery or management delay. Contrast‐enhanced ultrasound (CEUS), allowing for exclusive visualization of blood flow, might improve the diagnostic accuracy of adnexal torsion. Its feasibility and potential usefulness in this indication have recently been retrospectively assessed in a pediatric population, and the results confirmed high diagnostic performance.^14^, ^15^ However, this is the only retrospective study in the literature, and it is essential to confirm these data in a prospective and controlled study conducted in women.

The main objective of this study was to evaluate the diagnostic performance of CEUS, analyzed by experts and nonexperts, for improving diagnosis of adnexal torsion in women with acute abdominal pain and suspected adnexal torsion. As a secondary objective, we assessed agreement between several nonexpert obstetrician and gynecologists (ObGyn) to diagnose adnexal torsion based on CEUS imaging. Finally, we have described the potential impact of a new management strategy, including surgical decision based on CEUS results on avoided and missed surgery.

## 2 MATERIALS AND METHODS

This was a pilot study of the diagnostic accuracy of CEUS for the detection of adnexal torsion, conducted in a university hospital of obstetrics and gynecology in Nancy (France) between April 13, 2021 and April 16, 2022. The study was approved by the French Ethics Committee, the CPP (Comité de Protection des Personnes) OUEST I, on July 3, 2020, with reference number 2020T1‐16. CEUS was used in conjunction with abdominal surgery and, consequently, the study was registered as a single center, single‐arm trial on the ClinicalTrials.gov (NCT04522219) and EudraCT (2020‐000993‐27). The STARD (Standards for Reporting of Diagnostic Accuracy) guidelines were followed. The protocol has been previously published.^16^

All women with an emergency consultation for acute abdominal pain and who were scheduled for surgery for suspected adnexal torsion were eligible for inclusion. The other inclusion criteria were as follows: over 18 years old, affiliated with social security, complete information, and written consent. Exclusion criteria were women under measures of legal protection, with a contraindication to contrast injection (hypersensitivity to sulfur hexafluoride or any of the other ingredients, history of cardiac disease, respiratory distress syndrome, or severe pulmonary hypertension) or pregnancy.

**FIGURE 1:**
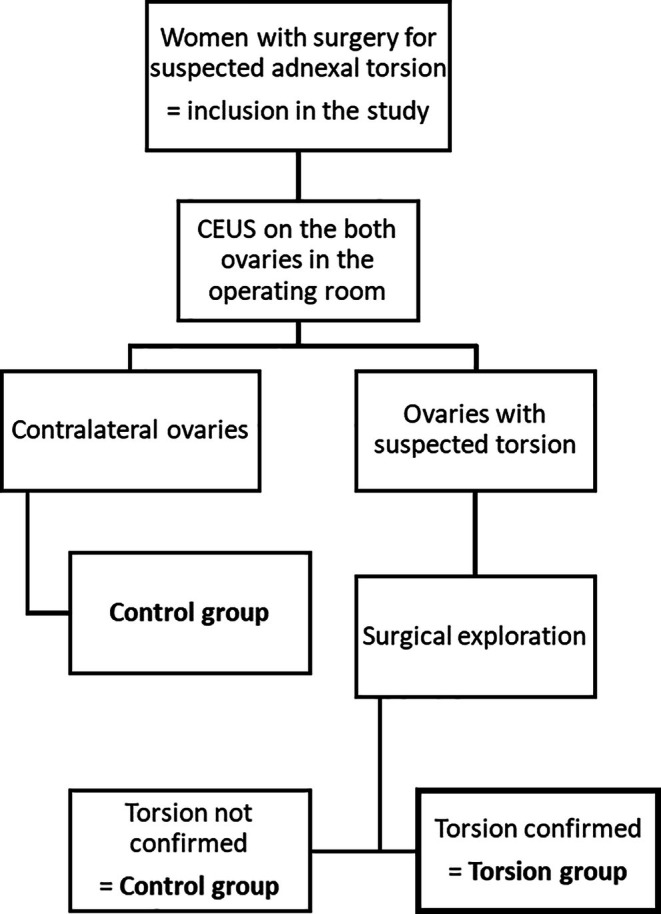
Workflow.

Contrast‐enhanced ultrasound was performed in the operating room just before induction for general anesthesia on each woman and on both ovaries if possible (ovary with suspected torsion and contralateral ovary). The unit of assessment was ovary, not woman, as both ovaries were evaluated in each woman. The two groups were compared according to the final diagnosis: the torsion group versus the control group. Ovaries were allocated to the groups based on surgical findings, considered the gold standard to confirm adnexal torsion. All contralateral ovaries in women who have undergone surgery were assigned to the control group as well as the ovaries with suspected but not confirmed adnexal torsion after the surgery. The ovaries complicated by surgically confirmed adnexal torsion were allocated to the torsion group. Therefore, the same woman can be allocated to the control group and the torsion group, depending on the ovary. The workflow of the allocation in groups is presented in Figure 1.

Ultrasound acquisition was performed with a HERA W10 (Samsung) and an endovaginal transducer (3–10 MHz). The ultrasound contrast product used was a Sonovue (BRACCO International B.V. Strawinskylaan 3051 NL‐1077 ZX Amsterdam Pays‐Bas); bolus injection at a volume of 2.4 mL of contrast product was administered, with a maximum of three injections per woman. Contrast‐enhanced acquisition was performed in contrast mode with standardized parameters, and a videoclip for 2 min was registered for each ovary. The image analysis of CEUS acquisition was qualitative and performed blindly in the final inclusion group. The qualitative analysis was performed by an ObGyn expert (C.B.), her expertise being defined by the previous works and publications on CEUS in other indications.^17^, ^18^ The analysis was then performed by ObGyn nonexperts, who are ObGyn without any formal training or knowledge in CEUS. They received an online questionnaire containing the anonymized videoclips of each case, without any data about clinical context and final diagnosis. They were asked to analyze imaging and classified as in torsion, no torsion, or uncertain. Their opinion about the utility of CEUS in the diagnosis strategy was also collected. The aim of this assessment was to show that qualitative image analysis was simple and feasible for ObGyn non‐experts unfamiliar with this imaging technique.

The primary endpoint was the presence or lack of ovary enhancement to assess the performance diagnosis of CEUS analyzed by an expert for detection of adnexal torsion. As secondary endpoints, we assessed the performance diagnosis of CEUS analyzed by nonexperts, the diagnosis accuracy of CEUS (percentage of correct diagnosis), the rate of uncertainty, the agreement between observers regarding confirmation of adnexal torsion, and the declared utility of CEUS in diagnosis strategy. The potential impact of a surgical decision based on CEUS was described by the assumed rate of surgery avoided (expected rate of false positives) or missed (expected rate or false negatives), calculated considering that the surgical decision was based on CEUS analysis only by a nonexpert ObGyn. In the event of an uncertain diagnosis in CEUS, we considered that surgical intervention would be performed.

The data collected were patient characteristics (medical history, history of abdominal surgery, body mass index), clinical symptoms (pelvic pain [type, irradiation, temporality, frequency, associated with nausea or vomiting, previous episode of pain, analgesic and morphine administration], clinical examination [abdominal palpation, vaginal examination]), ultrasound signs (ovaries sizes, presence of Doppler flow or ovarian cyst) and surgical findings to allocate groups (confirmed adnexal torsion, number of turns, color of the ovary before detorsion).

The sample size for this study was initially calculated for a quantitative analysis (reduced ovarian perfusion in the event of adnexal torsion) with a determination of a receiver operating characteristic curve and, with the lack of available data (no preliminary published data), was arbitrarily fixed at 30 women with an assumed distribution of 20 positive cases against 10 control cases with an accuracy of 5% and a power of 95% to have an area under the curve of 85% (R version 3.6.0, pROC package). When the CEUS was performed for the study, an on–off phenomenon was observed on the ovaries: in case of torsion, no enhancement at all was observed, which leads to a very efficient qualitative analysis. The quantitative analysis of enhancement was not relevant for clinical application. For this reason, the study was ended prematurely.^16^ Moreover, the sample size was sufficient to demonstrate the feasibility of CEUS in cases of adnexal torsion.

Characteristics including admission and clinical data of the women at inclusion in the two groups are expressed by the median, first, and third quartile for quantitative variables and the number and percentage for qualitative variables. They are compared using the χ^2^ or Fisher's exact test as appropriate for categorial variables and the Mann–Whitney test for quantitative variables.

Contrast enhanced ultrasound diagnostic performance was calculated according to the gold standard, defined by final diagnosis confirmed after surgery, by sensitivity, specificity, and their exact 95% confidence intervals, positive predictive value (PPV), negative predictive value (NPV), accuracy, positive and negative likelihood ratio (LR), and diagnosis odds ratio (DOR), as defined by the ratio LR+/LR−. Interrater agreement between all observers was assessed by Fleiss Kappa coefficient for multiple raters.

The overall alpha significance threshold was set at *P* < 0.05 in a bilateral situation. Statistical analysis was performed with R version 4.2.2.

## 3 RESULTS

**FIGURE 2:**
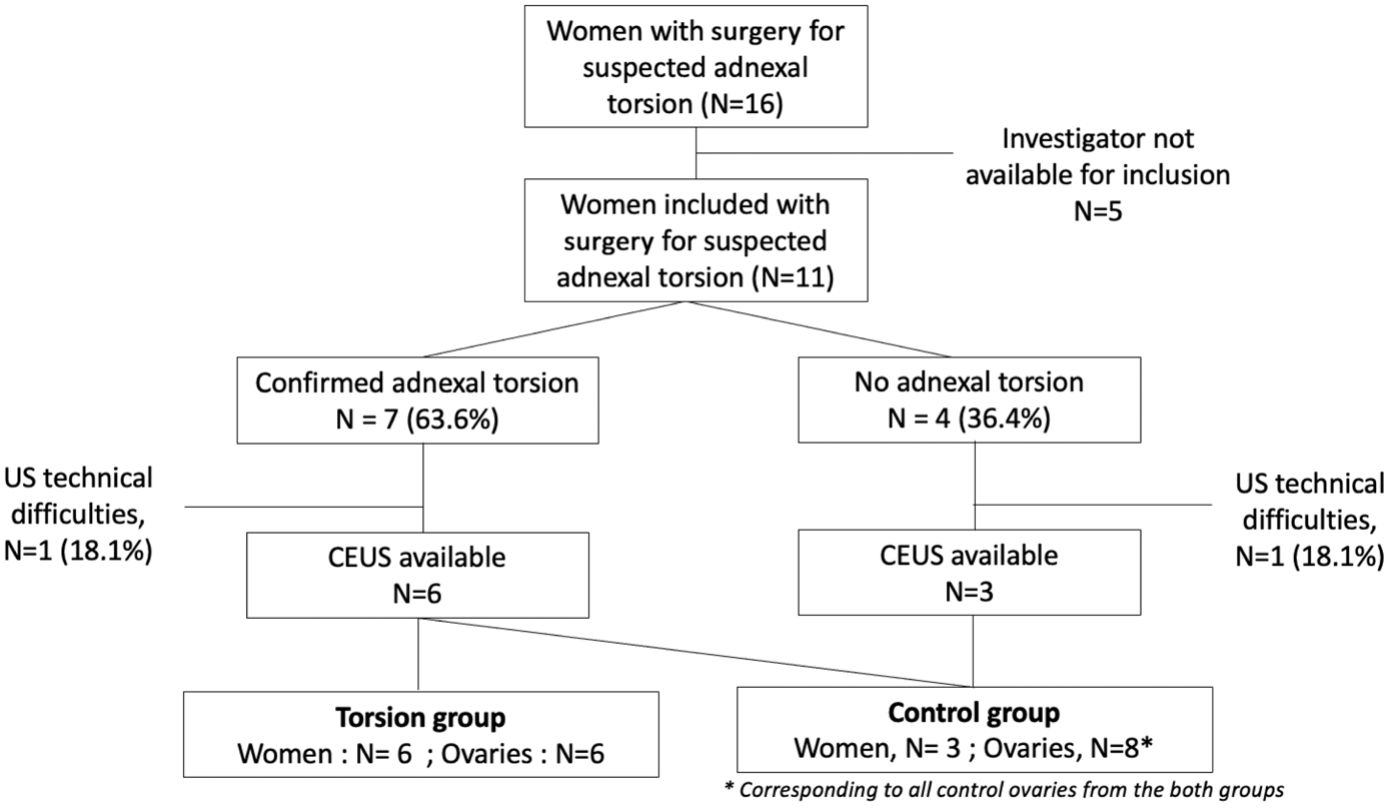
Flow chart.

During the study period, there were 16 eligible women with suspected adnexal torsion who underwent planned surgery. Among them, 11 (68.7%) women were included in the study: seven (63.6%) with confirmed adnexal torsion and four (36.4%) with unconfirmed adnexal torsion. Five women were not included because the investigator was not available for inclusion. CEUS data were available in 81.8% of cases (9/11 women), which corresponded to 14 cases divided into six ovaries in the torsion group and eight ovaries in the control group (Figure 2). These 14 cases were analyzed by nonexpert ObGyn via an online questionnaire for which we obtained 34 responses, divided into 20 ObGyn residents (58.8%) and 14 experienced ObGyn (41.2%) and which corresponds to a cohort of 476 CEUS images analyzed by nonexpert ObGyn.

The characteristics of the women and clinical data according to the allocated group for the suspected ovaries are described in Tables 1 and 2, respectively. There was no significant difference between the groups regarding clinical data at admission, except for body mass index, which was higher in women with unconfirmed adnexal torsion. All suspected ovaries without confirmation of adnexal torsion had an ovarian cyst, including one hemorrhagic cyst without pelvis effusion and one endometriotic cyst.

**TABLE 1:** Characteristics of women included according to the final diagnosis for the suspected ovary.

	Women with confirmed adnexal torsion (*N* = 7)	Women without confirmed adnexal torsion (*N* = 4)	*P*
Body mass index, median (Q1–Q3)	20.3 (18.8–20.7)	29.9 (23.5–37.3)	0.006^b^
History of pelvis surgery, *n* (%)	4 (57)	1 (25)	0.545
History of ovarian torsion, *n* (%)	0	0	NA
History of ovarian cyst, *n* (%)	3 (43)	3 (75)	0.545^a^
History of adnexectomy, *n* (%)	4 (57)	1 (25)	0.545^a^
History of surgery for ovarian cyst, *n* (%)	1 (14)	1 (25)	0.758^a^

Abbreviations: NA, not applicable; Q1, first quartile; Q3, third quartile.

^a^ Fisher exact test.

^b^ Mann–Whitney test.

**TABLE 2:** Clinical data according to the final diagnosis for the suspected ovary.

	Women with confirmed adnexal torsion (*N* = 7 women)	Women without confirmed adnexal torsion (*N* = 4 women)	*P*
Pelvic pain, *n* (%)			
Lateralized	7 (100)	4 (100)	NA
Radiating	3 (43)	1 (25)	1^a^
Onset of pain, *n* (%)			
Acute	5 (71)	4 (100)	0.491^a^
Gradual	2 (29)	0	
Frequency, *n* (%)			
Constant	6 (86)	4 (100)	1^a^
Intermittent	1 (14)	0	
Associated signs (nausea, vomiting), *n* (%)	5 (71)	2 (50)	0.576^a^
History of similar pain, *n* (%)	4 (57)	1 (25)	0.545^a^
Abdominal palpation, *n* (%)			
Elective pain	7 (100)	4 (100)	1^a^
Diffuse pain (defense)	2 (29)	2 (50)	0.576^a^
Elective pain at vaginal examination, *n* (%)	2 (29)	3 (75)	0.273^a^
Evaluation of pain (numerical scale), median (Q1–Q3)	8 (4.5 9.5)	6.5 (5–8.5)	1^b^
Analgesic administration, *n* (%)	7 (100)	4 (100)	1^a^
Morphine administration, *n* (%)	3 (43)	0	0.236^a^
Place of ultrasound, *n* (%)			
ObGyn emergency	5 (71)	4 (100)	0.491^a^
Radiology department	2 (29)	0	
Presence of ovarian cyst, *n* (%)	4 (57)	3 (75)	1^a^
Presence of Doppler flow, *n* (%)	1 (14)	3 (75)	0.194^a^
Ovary size, median (Q1–Q3)			
Width	50 (39–51)	37 (34.5–40)	0.629^b^
Length	70 (65, 81)	49.5 (42.5–54)	0.065^b^
Ovary appearance			
Normal	1 (14)	4 (100)	NA
Necrotic	1 (14)	/	
Purplish	5 (71)	/	
Number of turns, median (Q1–Q3)	4 (2.5–4)	NA	NA

Abbreviations: NA, not applicable; Q1, first quartile; Q3, third quartile.

^a^ Fisher exact test.

^b^ Mann–Whitney test.

**FIGURE 3:**
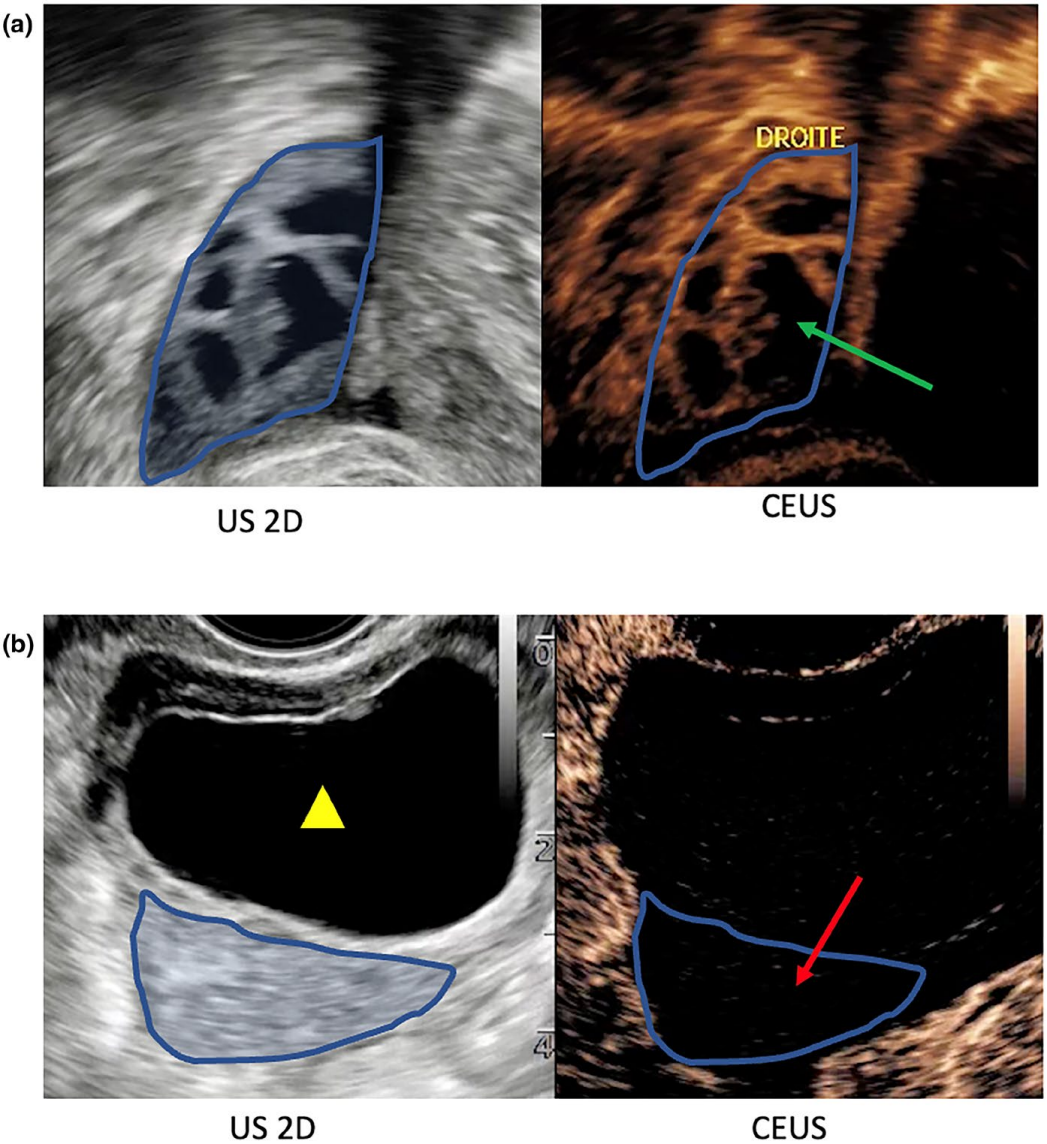
Contrast‐enhanced ultrasound in a control case (a) and ovarian torsion case (b). Transvaginal ultrasound in axial plan in two‐dimensional (2D) and contrast mode. In the 2D ultrasound image (US) (on the left), the ovarian parenchyma is outlined in blue, and the ovarian cyst is outlined by a yellow triangle. In the contrast image (CEUS) (on the right), we can see enhancement (green arrow) or lack of enhancement of the ovarian parenchyma (red arrow).

Contrast enhancement in the ovaries, evaluated qualitatively by an expert ObGyn, was never observed in the torsion group and always observed in the control group except in only one ovary (1/8) due to acquisition performed on a dermoid cyst instead of the ovarian parenchyma (Figure 3 and Table 3). For nonexpert ObGyn analyses, the diagnosis was correct in 78.4% of cases (373/476) and doubtful in 9.24% (44/476) of cases, more frequently for confirmed cases than control cases (16.2% vs. 4.1%; Table 4). Thus, the sensitivity and specificity were 100% (CI 95% [59–100]) and 86% (CI 95% [42–100]), respectively, for the qualitative analysis performed by the expert and 84.3% (CI 95% [78.7–88.9]) and 89.8% (CI 95% [85.4–93.2]), respectively, for the qualitative analysis performed by nonexperts (Table 5). There was strong agreement between observers to confirm lack of ovarian torsion (kappa coefficient 0.635) and moderate agreement to confirm the presence of adnexal torsion (kappa coefficient 0.459). Agreement was not different between the resident and experienced ObGyn. Ninety‐four percent of observers (32/34) declared that CEUS seemed useful in the diagnostic strategy in cases of suspected adnexal torsion.

**TABLE 3:** Cross‐tabulation of diagnosis by CEUS (analyzed by an expert) and that confirmed by surgery (gold standard).

Diagnosis by CEUS	Diagnosis by surgery		
	Torsion *N* = 6	No torsion *N* = 8	Total
Torsion (lack of enhancement)	6 (100%)	1 (12.5%)	7
No torsion (enhancement of the ovary)	0	7 (87.5%)	7
Total	6	8	14

Abbreviation: CEUS, contrast‐enhanced ultrasound.

**TABLE 4:** Cross‐tabulation of diagnosis by CEUS (analyzed by a nonexpert) and that confirmed by surgery (gold standard).

Diagnosis by CEUS	Diagnosis by surgery		
	Torsion	No torsion	Total
Torsion (lack of enhancement)	145 (71.1%)	33 (12.1%)	178
No torsion (enhancement of the ovary)	26 (12.7%)	228 (83.8%)	254
Doubtful	33 (16.2%)	11 (4.1%)	44
Total	204	272	476^a^

Abbreviations: CEUS, contrast‐enhanced ultrasound; ObGyn, obstetrician and gynecologist.

^a^ Total number of responses (14 cases analyzed by 34 ObGyn nonexpert: 14 * 34 = 476).

**TABLE 5:** Performance diagnosis of contrast‐enhanced ultrasound in ovarian torsion by expert and nonexpert observers.

	Value [95% CI]	
	Expert	Non expert
Sensitivity	100% [59–100]	84.3% [78.7–88.9]
Specificity	86% [42–100]	89.8% [85.4–93.2]
Positive predictive value	88% [47–100]	87.2% [81.9–91.5]
Negative predictive value	100% [54–100]	87.4% [82.7–91.3]
Positive likelihood ratio	7 [1.14–42.97]	8.24 [5.7–11.9]
Negative likelihood ratio	0 [0, NA]	0.17 [0.13–0.24]
Diagnosis odds ratio	7 [0, NA]	48.5 [43.8–49.6]

Abbreviations: CI, confidence interval; NA, not applicable.

If the surgical decision had been based only on the result of CEUS analyzed by an ObGyn nonexpert, 53.8% of women would avoid surgery with 16.2% of false positives and 12.7% false negatives. Among interventions not performed, 89.7% (228/254) would be unnecessary surgical interventions and 10.3% (26/254) would be useful interventions (Table 6).

**TABLE 6:** Cross tabulation comparing the surgical decision (based only on the result of CEUS analyzed by an ObGyn nonexpert) and diagnosis confirmed by surgery (gold standard).

Surgical decision based on CEUS result	Surgical diagnosis		
	Torsion	No torsion	Total
Surgery (torsion case and doubtful cases)	178/204 (87.3%)	44/272 (16.2%)	222 (46.6%)
No surgery (no torsion cases)	26/204 (12.7%)	228/272 (83.8%)	254 (53.4%)
Total	204 (42.8%)	272 (57.2%)	476

Abbreviations: CEUS, contrast‐enhanced ultrasound; ObGyn, obstetrician and gynecologist.

## 4 DISCUSSION

### 4.1 Principal findings

The diagnostic performance of CEUS in cases of suspected adnexal torsion was high, including for nonexpert ObGyn, with 84.3% sensitivity, 89.8% specificity, and a 48.5% diagnosis odds ratio. There was a strong agreement between nonexpert ObGyn to confirm the lack of adnexal torsion.

Our results first confirm the relevance of the question and need to improve the diagnostic performance of adnexal torsion because the false‐positive rate reached 30% in our cohort, which is consistent with the literature.^9^, ^10^, ^11^ Garde et al. (2023) evaluated the diagnostic accuracy of various ultrasound signs for detecting adnexal torsion in a systematic review and meta‐analysis.^6^ In that study, the sensitivity only reached 69% in cases of adnexal mass, and the specificity reached 95% in cases of ovarian Doppler flow presence. The best diagnosis odds ratio was 22 for the Doppler flow sign. If we compare our results to those data, CEUS seems to have a higher diagnostic performance, with a diagnosis odds ratio of 48.5 and sensitivity of 84.3%. However, the specificity of CEUS is similar to that of standard ultrasound.

Our results are also consistent with those of Trinci et al., who evaluated CEUS in a retrospective study with a similar sample size but in a pediatric population.^14^ They found a higher sensitivity (94.1%) and specificity (100%) than in our study, which can be explained by an evaluation exclusively performed by an expert and during dynamic acquisition, permitting a better analysis.

### 4.2 Clinical implications

Regarding imaging acquisition, CEUS is a simple imaging technique to use, with 81.8% (9/11) acquisition success in our study, which could reach near 100% in clinical practice. In fact, 20% of failures in our study corresponded to unavailability of the contrast module on the ultrasound machine because the software was not updated in the machine that was used exclusively for the study. Moreover, CEUS acquisition on ovaries is similar to a standard pelvic endovaginal ultrasound and, therefore, feasible by any ObGyn on duty.

However, the performance diagnosis could be improved by specific learning and dynamic analysis of images. In fact, in our study, image analysis was based on a videoclip, for which the recording was fixed on a single frame and assessed retrospectively. However, it is accepted that ultrasound is easier to interpret if the analysis is dynamic and performed directly by the operator. In addition, this point is most likely the reason for the only error made by the expert in retrospective analysis of images.

Finally, if the management strategy included a surgical decision based on CEUS results, one of two surgeries would be avoided. Among interventions not performed, 89.7% would be avoided interventions, but 10.3% would be missed interventions. Presumably, CEUS can reduce the false‐positive rate if integrated into the diagnostic management strategy. In fact, considering the 30% false‐positive rate in our cohort, it would have been reduced to 16% if the diagnosis had been based on the CEUS exam, a reduction of almost 50% of unnecessary surgery. Conversely, only one woman with adnexal torsion might not have had surgery, but it is likely that this error would not have occurred if the ultrasound analysis had been dynamic. We cannot draw a conclusion about the risk of false negatives associated with contrast ultrasound according to our study.

### 4.3 Research implications

Interventional studies are needed to evaluate the usefulness of CEUS to improve the management of women with suspected adnexal torsion for reducing both unnecessary emergency surgery and management delay.

### 4.4 Strengths and limitations

The strength of our study is that this is, to our knowledge, the first prospective study evaluating the diagnostic performance of CEUS in adnexal torsion in adults. Additionally, CEUS was analyzed both by an expert in CEUS and by several ObGyn without any formation in CEUS. This point reflects the immediate applicability in clinical practice because even in the absence of training, the diagnostic performance was high. The ease of use by nonexpert ObGyn reflect easy translation of this diagnostic service. The main limitation of our study was the small sample size and the study population, which included only cases with high clinical suspicion. Moreover, control healthy ovaries were included in the control group, but the lack of ovarian cysts in these cases can make CEUS analysis easier, both by expert and nonexpert ObGyn, and the performance diagnosis could be overestimated. Women were not analyzed as units (no comparison between them). All women are operated on, and all ovaries are analyzed. Each ovary is analyzed independently and not matched to the woman, as the analysis is qualitative. In fact, in qualitative assessment, there is no reason to have an absence of blood flow on an untwisted ovary. A hierarchy would have been relevant if the analysis had been quantitative on ovarian perfusion.

## 5 CONCLUSION

In conclusion, CEUS is a new imaging tool that is easy to implement, with clinical applicability and a diagnostic performance that appears to be higher than that of standard ultrasound.

## AUTHOR CONTRIBUTIONS

C.B., M.B, A.C, and O.M. designed the study; C.B. and A.L.F. wrote the manuscript and collected the data; G.H did the statistical analysis, and all authors reviewed and contributed to the manuscript.

## FUNDING INFORMATION

The study was supported by a grant from the French Ministry of Health (APJ 2019, N07).

## CONFLICT OF INTEREST STATEMENT

None.

## Data Availability

Research data are not shared.
